# Respiratory System Compliance Predicts Outcome After Lung Transplantation—A Retrospective Single Center Study

**DOI:** 10.3390/jcm14196941

**Published:** 2025-09-30

**Authors:** Cecilia Veraar, Stefan Schwarz, Peter Wohlrab, Johannes Geilen, Arabella Fischer, Thomas Neugebauer, Caroline Hillebrand, Bernhard Moser, Konrad Hoetzenecker, Martin Dworschak, Marcus J. Schultz, Edda M. Tschernko

**Affiliations:** 1 Division of Cardiac Thoracic Vascular Anesthesia and Intensive Care Medicine, Department of Anesthesiology, Intensive Care Medicine and Pain Medicine, Medical University of Vienna, 1090 Vienna, Austria; 2 Department of Thoracic Surgery, Medical University of Vienna, 1090 Vienna, Austria; 3 Department of Intensive Care Medicine, Amsterdam University Medical Center, 1007 MB Amsterdam, The Netherlands

**Keywords:** lung transplantation, respiratory system compliance, outcome, mechanical power

## Abstract

**Background:** Outcome prediction in patients undergoing lung transplantation (LUTX) for end-stage pulmonary disease can be challenging. We examined the prognostic value for mortality of respiratory system compliance (C_RS_) and mechanical power of ventilation (MP) at end of surgery in patients undergoing LUTX for end-stage pulmonary disease. **Methods:** In this single-center retrospective study, we included 755 patients undergoing LUTX between 2014 and 2023. The primary endpoint of this study was 1-year mortality, with 30-day mortality serving as a secondary endpoint. We conducted both univariate and multivariate analyses and constructed Receiver Operating Characteristic curves. **Results:** Of 755 patients, 1.9% and 12.2% patients died within 30 days and 1 year after LUTX. Fifteen-point four percent of all patients required extracorporeal membrane oxygenation (ECMO) prolongation into the early postoperative period. C_RS,_ but not MP was higher in 1-year survivors compared to non-survivors [median 25.8 mL/cmH_2_O (20.1, 32.1) and 22.5 mL/cmH_2_O (15.2, 28.4); *p* < 0.001] and [median 10.0 J/min (7.8, 12.0) and 9.3 J/min (6.2, 13.1); *p* = 0.329]. Moreover, low C_RS_ < 25.1 mL/cmH_2_O remained an independent factor for increased 1-year mortality after LUTX. Additionally, increased MP and C_RS_ were predictive for 30-day survival with an acceptable area under the curve of 0.758 (95% CI: 0.6–0.8; *p* < 0.001) and 0.735 (95% CI: 0.5–0.9; *p* = 0.003), and a sensitivity and specificity of 51% and 75.5% for MP and 50% and 85% for C_RS_, respectively. **Conclusions:** Postoperative C_RS_ serves as a significant independent predictor for short and long-term outcome in patients undergoing LUTX with and without ECMO prolongation into the early postoperative period.

## 1. Introduction

In the context of lung transplantation (LUTX), early prognostication of short-term and long-term outcomes is critical. Currently, prognostication remains complex and necessitates the integration of various clinical markers [1]. An ideal prognostic marker for both short- and long-term outcomes following LUTX should be easily determinable, robust, devoid of confounding factors, and exhibit a strong correlation with actual overall short- and long-term outcomes after transplantation [2,3].

Currently, primary graft dysfunction (PGD) assessment serves as a prognostic tool in order to predict early and late postoperative outcomes, including the development of chronic lung allograft dysfunction (CLAD) and long-term survival [1,2]. The International Society for Heart and Lung Transplantation (ISHLT) introduced a PGD scoring system, aligning it with the Berlin criteria for acute respiratory distress syndrome (ARDS) [4].

However, the use of the PGD score has several limitations: The radiographic scoring of PGD varies substantially between observers [5,6]. Furthermore, the P_a_O_2_/F_i_O_2_ ratio cannot reliably be assessed in patients requiring postoperative extracorporeal membrane oxygenation (ECMO) due to the dual source of blood oxygenation [6,7,8]. Consequently, there is a need for reliable and straightforward early prognostic parameters.

In ARDS patients, respiratory system compliance (C_RS_) has been shown to hold prognostic value, both in patients with and without ECMO support [9,10]. Studies have consistently demonstrated its utility in assessing lung function and guiding ventilatory management strategies [11]. C_RS_ is determined by a simple calculation of immediately available parameters during mechanical ventilation such as driving pressure (ΔP) and tidal volume (V_T_). In addition to C_RS_, the mechanical power of ventilation (MP) has recently been introduced as a prognostic parameter for outcomes after ARDS as well [12,13].

Therefore, we tested the hypothesis that C_RS_ and MP at the end of surgery have prognostic value in LUTX patients regarding 30-day and 1-year survival. For this purpose, we retrospectively analyzed a cohort of 755 consecutive patients who underwent LUTX for end-stage pulmonary disease at a high-volume lung transplantation center.

## 2. Methods

### 2.1. Design

This is a single center cohort study in patients who have been undergoing LUTX at the General Hospital of Vienna between January 2014 and April 2023. This study was conducted in accordance with the Declaration of Helsinki (as revised in 2013) and the study protocol was approved by the Ethics Committee of the Medical University of Vienna (1501/2023). The need for individual informed consent was waived due to the observational and retrospective nature of this study.

### 2.2. Patients

Patients were eligible if (1) aged > 18 years; and (2) if they underwent LUTX for end-stage pulmonary disease. We excluded patients with single-lung transplantation, as well as patients having multiorgan or re-transplantations.

### 2.3. Data Collection

Demographic and clinical data were retrieved from our intensive care data management system archives (IntelliSpace Critical Care and Anesthesia, Philips, Bollingen, Germany). Respiratory parameters for calculating MP and C_RS_ were collected at two distinct time points: baseline values (C_RS_1 and MP1) within the first 15 min after surgical incision and during the final 15 min before the end of surgery (C_RS_2 and MP2), i.e., after chest closure. Muscle relaxation was administered routinely before the skin incision and again before the end of surgery for subsequent tube exchange and bronchoscopy. Thus, C_RS_ and MP used for the statistical analysis were routinely determined during muscle relaxation. Ventilatory settings during LUTX adhered strictly to institutional standards, utilizing exclusively pressure control ventilation. Tidal volume (V_T_) targets ranged from 6 to 8 mL/predicted body weight (PBW), with a maximum inspiratory peak pressure (P_max_) < 30 cmH_2_O and a positive end-expiratory pressure [14] maintained at a minimum of 7 cmH_2_O. Lung-protective ventilation strategies were applied whenever feasible [11].

The following data were included for further analysis: (i) patient demographics and baseline characteristics including age, sex, body height, body weight, medical history, indications for LUTX, perioperative ECMO use, perioperative fluid balance, and perioperative transfusion of fresh frozen plasma (FFP) and packed red blood cells (PRBCs); (ii) perioperative ventilatory settings (i.e., tidal volume (V_T_), PEEP, P_max_, set and respiratory rate (RR) at the beginning and the end of the surgery, (iii) survival on day 30 and at 1 year post LUTX, duration of postoperative invasive ventilation, length of ICU and hospital stay; and (iv.) occurrence as well as degree of PGD.

PGD was graded as described elsewhere [2,5]. Time point T0 for PGD evaluation was 2 h after the patient’s arrival in the ICU, once their respiration and hemodynamics had stabilized after transfer from the operating room (OR). Subsequent time points T24, T48, and T72 were set 24, 48, and 72 h after admission to the ICU, respectively.

### 2.4. Perioperative Management

Anesthesia and surgery were performed according to our institutional standards as reported elsewhere [3]. LUTX was conducted either supported by intraoperative central veno-arterial-extracorporeal membrane oxygenation (VA-ECMO), the cardiopulmonary bypass, or without extracorporeal circulatory support [2,3,7]. If feasible, the patient was weaned from central VA-ECMO after implant of both lungs and adequate hemostasis at stable and sufficient gas exchange and hemodynamics. Prolongation of VA-ECMO support was chosen in response to the following conditions: PaO_2_/FiO_2_ ratio < 100, mean pulmonary arterial pressure > 2/3 of mean systolic arterial pressure, pronounced deterioration of respiratory and/or hemodynamic function. Therefore, central VA-ECMO was switched to a peripheral femoro-femoral VA-ECMO configuration [15].

### 2.5. Definitions and Calculations

V_T_ was expressed in mL/kg PBW with PBW being calculated using the Devine’s equations for females (1) and males (2), respectively:

PBW = 45.5 + 0.90 × (height in cm − 152.4),(1)

PBW = 50.0 + 0.90 × (height in cm − 152.4).(2)

C_RS_, driving pressure (ΔP), and MP were calculated during pressure-controlled ventilation using the following Equations (3)–(5):

C_RS_ = V_T_/ΔP, (3)

ΔP = Pmax − PEEP,(4)

MP = 0.098 × V_T_ × RR × (Pmax − 0.5 × ΔP). (5)

### 2.6. Study Endpoints

Endpoints of this analysis were short- (i.e., 30-day) and long-term (i.e., 1-year) survival.

### 2.7. Statistical Analysis

Normally distributed data are reported as means ± SD, non-normally distributed data as medians with interquartile range (IQR). Mann–Whitney U test was used to compare non-normally distributed data and *t*-tests to compare parametric data. Pearson’s correlation coefficient r was employed to estimate the strength of a linear relationship between respiratory parameters and clinical variables, such as the number of transfused PRBCs and FFPs, the highest arterial lactate level, the lowest hemoglobin level, length of invasive ventilation, and duration of ICU stay (Supplementary Materials Table S1).

We performed univariate and multivariate Cox regression analyses for 30-day and 1-year mortality. The median C_RS_ and the median MP were used as a cutoff to create two groups. Variables with *p* < 0.05 in the univariate analysis were entered into the multivariate model. We did not include blood hemoglobin values (HB) because of co-linearity with the number of transfused PRBCs.

Consecutively, we constructed Receiver Operating Characteristic (ROC) curves to present additional continuous data and calculated the respective areas under the curve (AUROC) for C_RS_ and MP to assess their predictive capability for short- and long-term survival. We plotted ROC curves for patients with and without ECMO prolongation separately. An AUROC between 0.5 and 0.7 was interpreted as poorly predictive for survival; 0.7 < AUROC < 0.8 as well; 0.8 < AUROC < 0.9 as very well, and 0.9 to 1.0 as excellently predictive. The median of C_RS_ and MP were used to calculate the sensitivity (True positive (TP)/(TP + false negative (FN)), the specificity (true negative (TN)/(TN + false positive (FP)), the positive predictive value (TP/(TP + FP)) and the negative predictive value (TN/(TN + FN)) to assess the performance of these parameters.

Statistical analyses and the corresponding graphical depiction were performed using SPSS software (version 28; IBM SPSS Inc., Chicago, IL, USA) and GraphPad Prism 9 (GraphPad Software, La Jolla, CA, USA). A *p* < 0.05 was considered statistically significant.

## 3. Results

### 3.1. Clinical and Demographic Data

Demographic data and perioperative characteristics are given in Table 1. We included 755 patients who underwent primary double LUTX. Overall, 17 (1.9%) and 92 (12.2%) patients died within 30 days and 1 year after LUTX, respectively.

**Figure 1:**
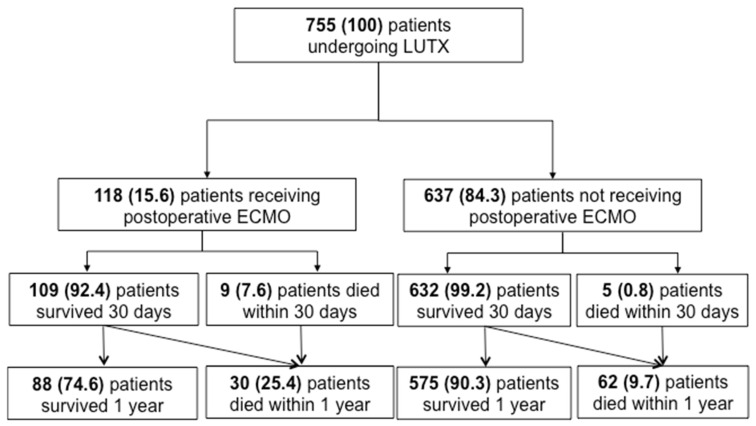
CONSORT Statement of patients undergoing LUTX with and without ECMO prolongation n this study, we included 755 patients who underwent orthotopic primary double LUTX. 118 patients required ECMO support in the postoperative period, and 637 did not require prolonged ECMO support into the postoperative phase. Five and 9 patients with and without ECMO support died within 30 days after LUTX. Moreover, 30 and 62 patients died within 1 year after transplantation with and without ECMO support, respectively.

At T0, 494 patients (65.4%) were classified as PGD grade 0, 30 patients (4.0%) as PGD grade 1, 40 patients (5.3%) as PGD grade 2, 91 patients (12.1%) as PGD grade 3, 84 patients (11.1%) were categorized as PGD ungradable and data from 16 patients (2.1%) was not available. The CONSORT statement of patients who underwent LUTX with and without ECMO prolongation is shown in Figure 1. In total, 118 patients required VA-ECMO support following LUTX. Among these patients, the 1-year mortality rate was 25% (*n* = 30). In contrast, 637 patients did not require postoperative ECMO support, with a 1-year mortality rate of 9% (*n* = 62).

### 3.2. C_RS_ and MP in Survivors and Non-Survivors

**Figure 2:**
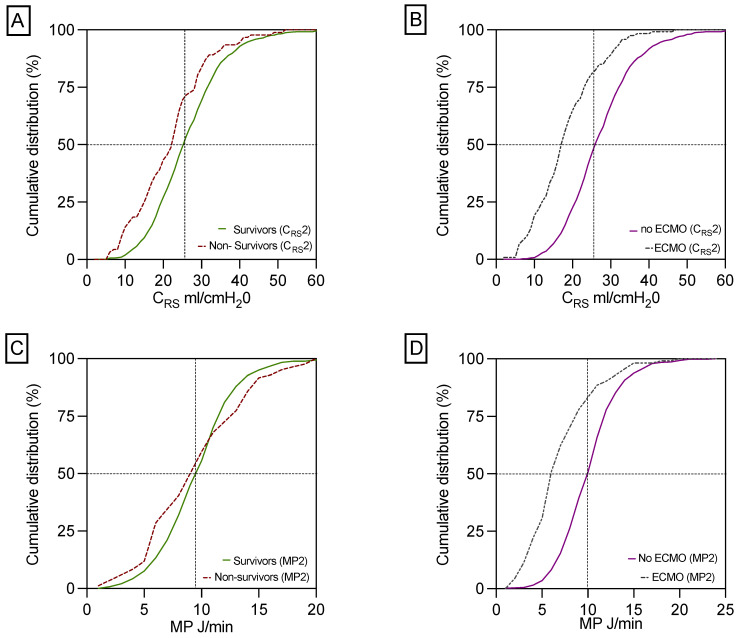
Differences in C_RS_ and MP between 1-year survivors and non-survivors and in patients with and without postoperative ECMO support. This panel shows the significant difference in C_RS_ between 1-year survivors and non-survivors (**A**). C_RS_ is decreased in patients with ECMO prolongation compared to patients without ECMO support (**B**). MP did not differ statistically significantly between 1-year survivors compared to 1-year non-survivors (**C**). In contrast, patients without ECMO prolongation had a significantly increased MP compared to patients on ECMO support (**D**). Respiratory parameters for calculating MP2 and C_RS_2 were collected during the final 15 min before the end of surgery, i.e., after chest closure.

1-year survivors exhibited significantly better C_RS_, but not MP, post- LUTX in contrast to non-survivors, as illustrated in Table 1 and Figure 2A,C. This association retained statistical significance for C_RS_ in patients with and without post-transplant VA-ECMO as delineated in Table 2. Furthermore, C_RS_ was markedly decreased in ECMO-patients compared to those who did not need ECMO support post-transplant (Figure 2B), while MP was significantly reduced in postoperative ECMO patients relative to their non-ECMO counterparts (Figure 2C). The correlations between clinical and respiratory parameters at the end of surgery are outlined in the Supplementary Materials Table S1.

### 3.3. Independent Predictors for Mortality in the Multivariate Analysis

C_RS_ 25.1 mL/cmH_2_O was an independent risk factor for 1-year mortality. Younger age (i.e., <61 years) was associated with a lower risk for 30-day, but not for 1-year mortality. Serum creatinine (sCR) levels > 1 mg/dL was another independent risk factor for 30-day and 1-year mortality. CTEPH was independently related to 30-day mortality. Serum lactate levels exceeding 3 mmol/L were also associated with 1-year mortality. Furthermore, transfusion of >16 PRBCs remained an independent predictor of 30-day and 1-year mortality, as shown in Table 3.

### 3.4. ROC Curves

**Figure 3:**
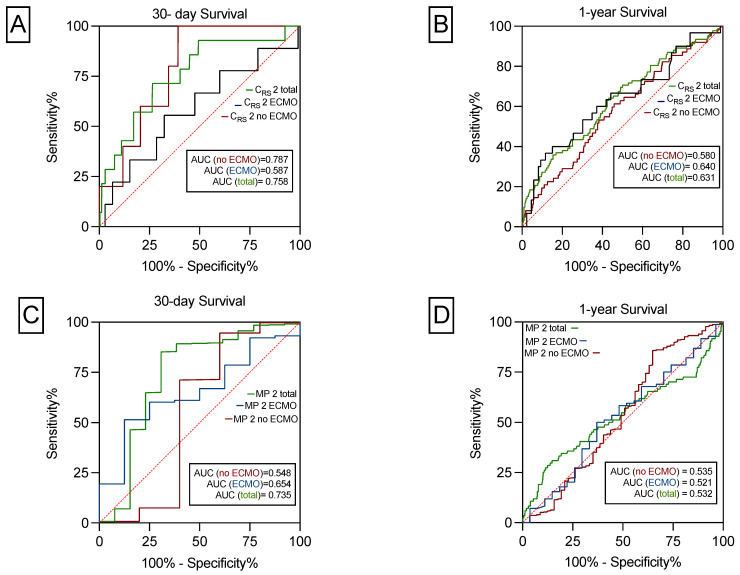
The predictive power of C_RS_ and MP on 30-day and 1-year survival. C_RS_ significantly predicts 30-day survival for the total cohort and for patients with and without ECMO (**A**). C_RS_ significantly predicts 1-year survival for the total cohort and for patients with, but not without ECMO (**B**). MP predicts 30-day survival for the total cohort, but not for patients with or without prolonged ECMO support (**C**). MP did not predict 1-year survival for the total cohort or for patients with and without postoperative ECMO use (**D**).

In all patients, the AUROC of C_RS_ for 30-day survival was 0.758 (95% CI: 0.6–0.8; *p* < 0.001). In patients without postoperative ECMO support it was 0.787 (95% CI: 0.6–0.9; *p* = 0.026) and in ECMO patients 0.587 (95% CI: 0.3–0.7; *p* = 0.386; Figure 3A).

In the total cohort, the AUROC of C_RS_ for 1-year survival was 0.631 (95% CI: 0.5–0.7; *p* < 0.001), in patients with postoperative ECMO it was 0.640 (95% CI: 0.5–0.7; *p* = 0.017), and in no-ECMO patients, 0.580 (95% CI: 0.5–0.6; *p* = 0.037) as depicted in Figure 3B.

As shown in Figure 3C, the AUROC of MP for 30-day survival was 0.735 (95% CI: 0.5–0.9; *p* = 0.003). In patients with postoperative ECMO prolongation, it was 0.654 (95% CI: 0.4–0.8; *p* = 0.147) and in patients without postoperative ECMO support 0.548 (95% CI: 0.1–0.9, *p* = 0.711). The AUROC of MP for 1-year survival was not statistically significant (Figure 3D). Sensitivity, specificity, NPV, PPV was shown for 30-day and 1-year survival for C_RS_, MP and PGD 0 ad depicted in Table 4. For calculations we used the median of C_RS_ of 25.1 mL/cmH_2_O and the median for MP of 9.9 J/min. C_RS_ and MP had the highest PPV of 99% to predict 30-day survival. The highest NPV of 15% was found for C_RS_ and no PGD to predict 1-year survival. Moreover, no PGD had the highest sensitivity of 67% to predict 30-day and 1-year survival. In contrast, the highest specificity of 85% was found for C_RS_ to predict 30-day survival.

## 4. Discussion

In this large-scale single center cohort study, our results underscore the pivotal role of C_RS_ at the end of surgery as a robust and easily available predictor not only for short-term (30-day) but also for long-term (1-year) survival following LUTX, regardless of postoperative ECMO support. Moreover, C_RS_ was an independent marker for 1-year mortality in the cox regression analysis. Despite the unclear influence of lung-protective ventilation on C_RS_ in ECMO patients, we, nevertheless, identified a similar association between C_RS_ and survival within this specific subgroup.

The predictive power of increased C_RS_ on 30-day survival appeared to be more pronounced in patients with lower disease severity, who did not require postoperative ECMO support. However, this distinction was no longer evident regarding prediction of 1-year survival. Conversely, reduced C_RS_ emerged as an independent marker for 1-year mortality in the Cox regression analysis. In addition, PPV and specificity to predict 30-day and 1 year survival was higher for C_RS_ as compared to PGD 0.

Significantly elevated C_RS_ at the end of LUTX in 1-year survivors reflected a correspondingly lower driving pressure (ΔP). This aligns with the findings of Amato et al., where decreased ΔP was positively associated with a survival benefit in ARDS patients [16]. Nonetheless, in 2012, the ARDS Berlin taskforce considered C_RS_ solely as an ancillary variable and did not include C_RS_ in its final definition of ARDS due to limited evidence supporting its predictive validity at that time [4].

Contemporary scoring systems designed to define both ARDS and PGD that ultimately predict outcome face challenges, particularly related to high inter-observer variations in the interpretation of chest images [6,8]. Moreover, interpretation of chest X-rays immediately post-LUTX is prone to various confounders such as bleeding and pleural effusion, which can mimic bilateral infiltrations [8]. Additionally, a key variable in PGD grading is P_a_O_2_/F_i_O_2_ ratio, which is substantially confounded in patients requiring postoperative ECMO treatment. These downsides of current PGD grading led to the introduction of the term “PGD ungradable” in ECMO-supported patients without diffuse infiltrates in their chest X-rays [15].

Our study revealed intriguing findings regarding the role of MP in predicting survival following LUTX. Surprisingly, increased MP at the end of LUTX was associated with better short-term survival (30 days), while reduced MP predicted higher than 30-day and 1-year mortality. This unexpected outcome diverges from the conventional understanding whereby higher MP typically signifies elevated stress on the respiratory system, potentially leading to adverse outcomes [17]. However, our study included patients with prolonged postoperative ECMO support, a group known to have a generally poorer prognosis. In these ECMO patients, we observed a reduced MP. This inverse relationship between MP and mortality in ECMO patients may be attributed to the application of lung-protective ventilation in patients on ECMO. These strategies aim to mitigate further lung injury and are known to lower MP [18]. Therefore, relying solely on MP as a predictive marker may be less advantageous in cohorts that include transplant recipients on ECMO. In contrast, a study focusing exclusively on ECMO patients suggested that MP, when corrected by C_RS_, was associated with increased mortality [19], emphasizing the importance of considering both factors in this particular subgroup.

Previous literature has reported that higher BMI is associated with reduced CRS, primarily due to the mechanical effects of adiposity on lung expansion and elastic properties. In our study, 1-year non-survivors had a slightly higher BMI, though this difference was not statistically significant. Similarly, we found no significant difference in CRS between patients with high and low BMI when using the median as a cut-off. Since a BMI > 35 kg/m^2^ is the standard upper limit for lung transplant candidacy in Europe, our cohort did not include obese patients, which may explain the absence of significant differences in respiratory compliance.

Our study has several limitations due to its retrospective nature and the single-center analysis of prospectively and automatically collected data. While the single-center approach ensures homogeneous patient management, the impact of different management strategies in other transplant centers remains unclear. Another limitation concerns surgical access, as patients transplanted via clamshell or via thoracotomy have been included where the surgical access could potentially affect postoperative C_RS_. However, to minimize procedural variability, we excluded complex patient groups, as those who underwent multiorgan transplantations and patients scheduled for re-transplantation. Additionally, we did not include patients undergoing single-LUTX due to the uncertain extent of the impact on lung function by the residual lung [5]. Another limitation of our study concerns the use of serum creatinine as a proxy for renal function, as eGFR was not available in our dataset; while we adjusted for age and gender, which influence creatinine levels, the absence of eGFR may limit the precision of renal function assessment.

Furthermore, due to the lack of a clinically defined cut-off value for C_RS_ that separates poor from acceptable compliance, we employed the median to dichotomize patients and to facilitate uni- and multivariate Cox regression analyses.

## 5. Conclusions

C_RS_ serves as a significant independent predictor marker to predict 30-day and 1-year survival, irrespective of continued postoperative ECMO support. MP should be interpreted with caution in cohorts post-LUTX that include ECMO patients due to its inverse relationship seen in this subgroup.

## Figures and Tables

**Table 1:** Clinical and demographic data. Clinical and patient characteristics of 1-year survivors and non-survivors.

	Total *n* = 755	Survivors *n* = 663	Non-Survivors *n* = 92	*p*-Value
**Demographic data**				
Female ^#^	346 (45.8)	308 (46.5)	38 (41.3)	0.382
Age (years) ^##^	50 ± 15.4	49.9 ± 15.4	50.9 ± 15.9	0.271
BMI (kg/m^2^) ^##^	22.4 ± 4.2	22.2 ± 4.2	23.0 ± 4.4	0.051
**Diagnosis**				
COPD ^#^	282 (37.4)	258 (38.9)	24 (26.1)	
ILD ^#^	185 (24.5)	153 (23.1)	32 (34.8)	
CF ^#^	125 (16.6)	112 (16.9)	13 (14.1)	
IPAH ^#^	52 (6.9)	45 (6.8)	7 (7.6)	
Alpha-1 antitrypsin deficiency ^#^	30 (4.0)	26 (3.9)	4 (4.3)	
COVID ARDS ^#^	27 (3.6)	18 (2.7)	9 (9.8)	
Miscellaneous ^#^	21 (2.8)	21 (3.2)	0 (0.0)	
Sarcoidosis ^#^	9 (1.2)	9 (1.4)	0 (0.0)	
LAM ^#^	7 (0.9)	6 (0.9)	1 (1.1)	
Histiozytosis ^#^	4 (0.5)	4 (0.6)	0 (0.0)	
Bronchiectasis ^#^	4 (0.5)	3 (0.5)	1 (1.1)	
CTEPH ^#^	3 (0.4)	2 (0.3)	1 (1.1)	
Alveolarproteinosis ^#^	3 (0.4)	3 (0.5)	0 (0.0)	
Post-COVID fibrosis ^#^	3 (0.4)	3 (0.5)	0 (0.0)	
**Perioperative data**				
Lactate max (mmol/L) ^##^	3.3 ± 1.5	3.2 ± 1.5	3.6 ± 1.9	**0.035**
HB min (g/dL) ^##^	9.2 ±1.3	9.2 ± 1.3	8.9 ± 1.5	0.091
PRBC *	8 (4.6, 13.3)	7.8 (4.0, 12.6)	9.0 (6.0, 13.0)	**<0.001**
FFP *	10 (6.0, 14.0)	9.0 (6.0, 13.0)	10.0 (6.9, 20,0)	**0.037**
Crystalloids (mL) *	1500 (1000, 2500)	1500 (1000, 2500)	1500 (1000, 2200)	0.199
**Extracorporeal circulation**				
CPB ^#^	10 (1.3)	9 (1.4)	1 (1.1)	0.306
preoperative ECMO ^#^	85 (11.3)	62 (9.4)	23 (25.0)	**<0.001**
intraoperative ECMO ^#^	738 (97.7)	647 (97.3)	91 (8.9)	0.631
postoperative ECMO ^#^	118 (15.6)	88 (13.3)	30 (32.6)	**<0.001**
no ECMO ^#^	7 (0.9)	7 (0.9)	-	0.116
**Respiratory parameters**				
*Before LuTX (native lung)*				
Pmax (cmH_2_O) *	24.1 (19.3, 30.5)	23.8 (19.0, 30.1)	27.1 (21.9, 35.0)	**<0.001**
PEEP (cmH_2_O) *	4.6 (3.3, 5.2)	4.6 (3.4, 5.1)	4.7 (3.2, 6.0)	0.328
ΔP (cmH_2_O) *	19.6 (15.0, (25.7)	19.2 (14.7, 25.5)	22.3 (16.5, 29.7)	**<0.001**
VT (mL/kg) *	6.8 (5.4, 8.3)	6.9 (5.5, 8.4)	6.4 (4.7,7.9)	0.375
RR (per min) *	14 (12, 16)	14 (12, 16)	15 (12, 17)	0.074
C_RS_1 (mL/cmH_2_O) *	21.9 (14.2, 32.6)	22.5 (14.9, 33.1)	17.7 (10.4, 24.8)	**<0.001**
MP1 (J/min) *	8.2 (5.9, 11.4)	8.2 (5.8, 11.1)	8.8 (6.4, 12.3)	0.158
*At the end of LuTX (transplanted lung)*				
Pmax (cmH_2_O) *	24.5 (22.1, 26.8)	24.2 (22.0, 26.5)	25.8 (23.1, 28.0)	**<0.001**
PEEP (cmH_2_O) *	7.0 (6.0, 8.0)	7.0 (5.8, 8.0)	7.0 (6.1, 8.0)	**0.028**
ΔP (cmH_2_O) *	17.6 (15.0, 20.3)	17.3 (14.8, 20.2)	18.1 (15.7, 21.2)	**0.034**
VT (mL/kg) *	6.9 (5.7, 8.2)	7.0 (5.8, 8.3)	6.0 (4.6, 7.4)	**<0.001**
RR (per min) *	15 (13, 16)	15 (14, 16)	15 (13, 18)	0.516
C_RS_2 (mL/cmH_2_O) *	25.1 (19.4, 31.5)	25.8 (20.1, 32.1)	22.5 (15.2, 28.4)	**<0.001**
MP2 (J/min) *	9.9 (7.7, 12.0)	10.0 (7.8, 12.0)	9.3 (6.2, 13.1)	0.329
***Outcome***				
*PGD time point 0 h*				
PGD 0	494 (65.4)	441 (66.5)	53 (57.6)	
PGD 1	30 (4.0)	27 (4.1)	3 (3.3)	
PGD 2	40 (5.3)	39 (5.9)	1 (1.1)	
PGD 3	91 (12.1)	75 (11.3)	15 (17.4)	
PGD ungradable	84 (11.1)	67 (10.1)	17 (18.5)	
PGD missing	16 (2.1)	14 (2.1)	2 (2.2)	**0.016**
*PGD time point 24 h*				
PGD 0	594 (72.7)	493 (74.4)	56 (60.9)	
PGD 1	53 (7.0)	50 (7.5)	3 (3.3)	
PGD 2	30 (4.0)	27 (4.1)	3 (3.3)	
PGD 3	33 (4.4)	22 (3.3)	11 (12.0)	
PGD ungradable	73 (9.7)	58 (8.7)	15 (16.3)	
PGD missing	17 (2.3)	13 (2.0)	4 (4.3)	**<0.001**
*Ventilation support and length of stay*				
Duration of MV (days)	7 (4, 16)	6 (4, 12)	9 (6, 18)	**<0.001**
ICU length of stay (days)	9 (6, 21)	9 (18, 18)	22 (9, 49)	**<0.001**
Hospital length of stay (days)	27 (20, 42)	27 (20, 40)	36 (23, 71)	**<0.001**

ARDS, acute respiratory distress syndrome; CF, cystic fibrosis; COPD, chronic obstructive pulmonary disease; CPB, cardiopulmonary bypass; CTEPH, chronic thromboembolic pulmonary hypertension; ECMO, extracorporeal membrane oxygenation; FFP, fresh frozen plasma; HB, hemoglobin; ILD, idiopathic lung disease; IPAH, idiopathic pulmonary arterial hypertension; LAM, Lymphangioleiomyomatosis; MP, mechanical power; MV, mechanical ventilation; *n*, number; PEEP, positive end-expiratory pressure; Pmax, maximum airway pressure; PRBCs, packed red blood cells; RR, respiratory rate; VT, tidal volume; ^#^ *n* (%), ^##^ mean ± SD, * median (IQR).

**Table 2:** Mechanical ventilation at the end of surgery.

*At End of TX*	No Postoperative ECMO			ECMO Prolongation		
	Survivors *n* = 592	Non-Survivors *n* = 64	*p*-Value	Survivor *n* = 89	Non-Survivor *n* = 33	*p*-Value
Pmax (cmH_2_O) *	24.1 (22.0, 26.2)	25.1 (22.2, 27.6)	0.056	25.0 (22.6, 28.0)	26.9 (24.8, 30.0)	**0.047**
PEEP (cmH_2_O) *	6.8 (5.6, 7.8)	6.9 (5.9, 7.7)	0.767	7.5 (6.1, 8.1)	8.0 (7.0, 9.9)	**0.040**
ΔP (cmH_2_O) *	17.3 (15.0, 20.1)	17.8 (15.2, 20.9)	0.130	17.5 (14.9, 20.9)	18.6 (16.0, 21.5)	0.459
VT (mL/kg) *	7.2 (6.1, 8.4)	6.9 (5.9, 7.7)	**0.011**	5.1 (4.0, 6.7)	4.6 (2.8, 6.5)	0.167
RR (per min) *	15 (14, 16)	15 (14, 18)	0.261	14 (11, 16)	14 (12, 18)	0.450
C_RS_ (mL/cmH_2_O) *	26.5 (21.2, 32.7)	24.1 (19.2, 30.3)	**0.037**	18.6 (13.6, 24.4)	15.8 (9.6, 23.0)	**0.034**
MP (J/min) *	10.4 (8.4, 12.1)	10.6 (8.2, 13.8)	0.382	6.8 (4.9, 9.1)	6.2 (4.5, 9.8)	0.741

C_RS_, respiratory compliance; MP, mechanical power; *n*, number; PEEP, positive end-expiratory pressure; Pmax, maximum airway pressure; RR, respiratory rate; VT, tidal volume; ΔP driving pressure; * median (IQR).

**Table 3:** Cox regression analysis of 30-day and 1-year mortality.

**A**		**Univariate Model**			**Multivariate Model**		
		**HR**	**CI 95%**	***p*-Value**	**HR**	**CI 95%**	***p*-Value**
**30-day all-cause mortality**							
**C_RS_**	≥25.1 mL/mbar	1.0					
	<25.1 mL/mbar	**6.0**	1.3–27.0	**0.005**	5.2	0.7–38.2	0.099
MP	≥9.9 J/min	1.0					
	<9.9 J/min	3.4	0.9–12.6	0.058			
Gender	male	1.0					
	female	1.8	0.6–5.1	0.257			
Age	≤20 years	1.0			1.0		
	21–40 years	0.6	0.1–2.5	0.489	**0.1**	0.0–0.7	**0.024**
	41–60 years	**0.1**	0.0–0.7	**0.018**	**0.0**	0.0–0.6	**0.022**
	61–75 years	0.2	0.0–1.1	0.068	0.1	0.0–2.1	0.168
sCR	≤1 mg/dL	1.0					
	1–2 mg/dL	**6.4**	2.1–19.1	**<0.001**	**4.1**	1.1–14.6	**0.027**
Diagnosis	COPD	1.0			1.0		
	ILD	**9.2**	1.1–76.5	**0.040**	2.8	0.2–2.0	0.586
	CF	6.7	0.7–65.8	0.093	0.5	0.0–13.6	0.686
	IPAH	**16.6**	1.7–160	0.015	0.8	0.0–18.0	0.918
	Alpha 1	0.0	0.0–	0.996	0.0	0.0–	0.993
	COVID ARDS	0.0	0.0	**<0.001**	0.0	0.0	0.998
	Miscellaneous	0.0	0.0–	0.992	0.0	0.0–	0.991
	Sarcoidosis	0.0	0.0–	0.989	0.0	0.0–	0.993
	LAM	0.0	0.0–	0.995	0.0	0.0–	0.997
	Histiozytosis	0.0	0.0–	0.996	0.0	0.0–	0.992
	Bronchiectasis	0.0	0.0–	**0.994**	0.0	0.0–	0.996
	CTEPH	**112**	7.0–1803	**<0.001**	**85.5**	2.6–319.0	**0.006**
	Alveolarproteinosis	0.0	0.0–	0.997	0.0	0.0–	0.997
	Post-COVID fibrosis	0.0	0.0–	0.990	0.0	0.0–	0.993
ECMO	postop ECMO (yes)	9.9	3.3–29.7	**<0.001**	1.8	0.4–8.1	0.168
	preop ECMO (yes)	2.1	0.6–7.2	0.239			
HB min	>9.2 g/dL	1.0					
	≤9.2 g/dL	1.8	0.7–6.2	0.253			
BL max	<3.0 mmol/L	1.0					
	3.0–6.0 mmol/L	2.1	0.6–7.0	0.219			
	>6.0 mmol/L	4.3	0.8–23.9	0.088			
PRBCs	<8 units	1.0			1.0		
	>8–16 units	6.2	0.7–56.0	0.097	3.8	0.4–37.4	0.241
	>16 units	**23.0**	2.8–184	**<0.001**	**9.7**	1.0–94.4	**0.050**
FFPs	<5 units	1.0					
	>5–10 units	0.4	0.0–6.4	0.522			
	>10–15 units	0.8	0.0–12.8	0.875			
	>15–20 units	0.0	0.0–	0.989			
	>20 units	5.9	0.6–56.8	0.124			
**B**		**Univariate Model**			**Multivariate Model**		
		**HR**	**CI 95%**	***p*-Value**	**HR**	**CI 95%**	***p*-Value**
**1-year all-cause mortality**							
**C_RS_**	≥25.1 mL/mbar	1.0					
	<25.1 mL/mbar	**1.9**	1.2–3.0	**0.002**	**2.1**	1.1–4.8	**0.048**
MP	≥9.9 J/min	1.0					
	<9.9 J/min	1.1	0.7–1.8	0.437			
Gender	male	1.0					
	female	1.1	0.7–1.7	0.417			
Age	≤20 years	1.0					
	21 < 40 years	0.6	0.2–1.5	0.367			
	41–60 years	0.6	0.3–1.4	0.332			
	61–75 years	1.0	0.4–2.2	0.887			
sCR	≤1 mg/dL	1.0					
	1–2 mg/dL	2.4	1.5–3.9	**<0.001**	**2.9**	1.3–6.5	**0.008**
Diagnosis	COPD	1.0			1.0		
	ILD	**2.0**	1.2–3.5	**0.006**	0.7	0.2–2.0	0.586
	CF	1.1	0.6–2.3	0.522	0.7	0.2–2.1	0.547
	IPAH	1.6	0.6–3.6	0.273	0.3	0.0–1.6	0.197
	Alpha 1	1.3	0.4–3.9	0.565	1.1	0.1–9.1	0.891
	COVID ARDS	**4.4**	**2.0–9.4**	**<0.001**	1.5	0.9–14.9	0.412
	Miscellaneous	0.0	0.0–2.4^e279^	0.971	0.0	0.0–	0.981
	Sarcoidosis	0.0	0.0–	0.981	0.0	0.0–	0.989
	LAM	1.7	0.2–12.8	0.588	2.1	0.2–18.0	0.490
	Histiozytosis	0.0	0.0–	0.986	0.0	0.0–	0.992
	Bronchiectasis	4.0	0.5–29.8	0.171	4.3	0.7–23.7	0.091
	CTEPH	5.0	0.6–37.6	0.111	3.1	0.3–24.9	0.279
	Alveolarproteinosis	0.0	0.0–	0.989	0.0	0.0–	0.981
	Post-COVID fibrosis	0.0	0.0–	0.991	0.0	0.0–	0.993
ECMO	postop ECMO (yes)	**3.1**	2.0–4.7	**<0.001**	1.2	0.5–2.9	0.592
	preop ECMO (yes)	2.8	1.7–4.5	**<0.001**	0.6	0.2–2.2	0.547
HB min	>9.2 g/dL	1.0					
	≤9.2 g/dL	**1.6**	1.1–2.5	**0.015**			
BL max	<3.0 mmol/L	1.0			1.0		
	3.0–6.0 mmol/L	1.2	0.8–1.8	0.316	**2.1**	1.0–4.6	**0.043**
	>6.0 mmol/L	**2.1**	1.0–4.3	**0.044**	1.1	0.3–4.2	0.786
PRBCs	<4 units	1.0			1.0		
	>4–8 units	2.2	0.7–6.5	0.135	2.2	0.4–10.2	0.296
	>8–16 units	**4.2**	1.4–11.8	**0.006**	**3.9**	0.7–20.2	0.107
	>16 units	**7.3**	2.6–20.9	**<0.001**	**7.1**	11–4.5	**0.027**
FFPs	<5 units	1.0			1.0		
	>5–10 units	0.7	0.2–1.7	0.446	1.0	0.3–3.0	0.867
	>10–15 units	0.5	0.2–1.6	0.327	0.5	0.1–1.6	0.270
	>15–20 units	1.2	0.4–3.8	0.693	0.7	0.2–2.7	0.692
	>20 units	**2.6**	1.0–6.7	**0.040**	0.7	0.2–2.6	0.668

BL, blood lactate concentration; FFP, fresh frozen plasma; HB min, minimum serum hemoglobin concentration; MP; mechanical power PRBCs, packed red blood cells; sCR, serum creatinine.

**Table 4:** Applicability of CRS, MP and no PGD to predict 30-day and 1-year survival.

	C_RS_ 30-Day Survival	C_RS_ 1-Year Survival	MP 30-Day Survival	MP 1-Year Survival	PGD (No) 30-Day Survival	PGD (No) 1-Year Survival
**PPV**	99%	91%	99%	88%	98%	89%
**NPV**	3%	15%	2%	10%	3%	15%
**Sensitivity**	50%	52%	51%	51%	67%	67%
**Specificity**	85%	65%	75%	52%	64%	41%

PPV, positive predictive value; NPV negative predictive value, PGD, primary graft dysfunction. 25.1 mL/cmH_2_O and the median for MP of 9.9 J/min.

## Data Availability

All data generated or analyzed during this study are included in this published article.

## Supplementary Materials

The supporting information can be downloaded at https://www.mdpi.com/article/10.3390/jcm14196941/s1. Table S1: Correlation of respiratory and clinical parameters.

## Abbreviations

ARDS	Acute Respiratory Distress Syndrome
C_RS_	Respiratory System Compliance
CLAD	Chronic Lung Allograft Dysfunction
ECMO	Extracorporeal Membrane Oxygenation
FFP	Fresh Frozen Plasma
FN	False negative
HB	Hemoglobin values
ISHLT	International Society for Heart and Lung Transplantation
IQR	Interquartile Range
LUTX	Lung Transplantation
MP	Mechanical Power of Ventilation
OR	Operating Room
PBW	Predicted Body Weight
PGD	Primary Graft Dysfunction
ΔP	Driving Pressure
PRBC	Packed Red Blood Cells
TN	True Negative
TP	True Positive
V_T_	Tidal Volume
RR	Respiratory Rate
ROC	Receiver Operating Characteristic
