# Supplementary material for: Respiratory System Compliance Predicts Outcome After Lung Transplantation—A Retrospective Single Center Study
Source: J Clin Med. 2025 Sep 30;14(19):6941. doi: 10.3390/jcm14196941 (PMC12525188; doi:10.3390/jcm14196941)
Supplement: Supplementary file 1 [file jcm-14-06941-s001.zip › jcm-3801201-supplementary.pdf]

Supplementary Materials

Respiratory System Compliance Predicts Outcome after Lung Transplantation–a retrospective single center study

Cecilia Veraar M.D, PhD[1], Stefan Schwarz M.D, PhD[2], Peter Wohlrab M.D[1], Johannes Geilen M.D[1], Arabella Fischer M.D, PhD[1], Thomas Neugebauer[1], Caroline Hillebrand. M.D[2], Bernhard Moser M.D, PhD, MBA[2], Konrad Hoetzenecker M.D, PhD, MBA[2], Martin Dworschak M.D, MBA[1], Marcus J. Schultz. M.D[1,3], Edda M. Tschernko M.D, MBA[1]

[1]Department of Anesthesiology, Intensive Care Medicine and Pain Medicine, Division of Cardiac Thoracic Vascular Anesthesia and Intensive Care Medicine, Medical University of Vienna, Vienna, Austria

[2]Department of Thoracic Surgery, Medical University of Vienna, Vienna, Austria

[3]Department of Intensive Care Medicine, Amsterdam University Medical Center, Amsterdam, The Netherlands

Correspondence to:

Cecilia Veraar, MD

Department of Anesthesia, General Intensive Care and Pain Management

Division of Cardiac-, Thoracic-, Vascular Anesthesia and Intensive Care Medicine

General Hospital Vienna, Medical University of Vienna

Währinger Gürtel 18–20

1090, Vienna, Austria

Email: cecilia.veraar@meduniwien.ac.at

RESULTS

Supplementary Table S1: Correlation of respiratory and clinical parameters

Respiratory parameters were measured during the last 15 minutes of LUTX.

	C_{RS}	MP	PΔ	RR	VT
**PRBC (count)**					
r	-0.253	-0.166	0.053	-0.072	-0.283
p-value	**<0.001**	**<0.001**	0.152	0.060	**<0.001**
**FFP (count)**					
r	-0.192	0.081	0.129*	0.092	-0.130*
p-value	**<0.0001**	0.135	**0.011**	0.088	**0.011**
**HB min (g/dL)**					
r	0.244	0.051	-0.099	-0.109	0.243
p-value	**<0.001**	0.183	**0.007**	**0.004**	, **<0.001**
**BL (mmol/L)**					
r	-0.203	-0.169	0.060	-0.027	-0.234
p-value	**<0.001**	**<0.001**	0.104	0.485	**<0.001**
**Duration of mechanical ventilation (days)**					
r	-0.248	-0.020	0.174	0.025	-0.191
p-value	**<0.001**	0.599	**<0.001**	0.506	**<0.001**
**ICU length of stay (days)**					
r	-0.279	-0.045	,167	0.056	-0.233
p-value	**<0.001**	0.236	**<0.001**	0.141	**<0.001**

BL, blood lactate concentration; FFP, fresh frozen plasma; Min HB, minimum serum hemoglobin concentration; MP, mechanical power; PRBCs, packed red blood cells; r, Pearson correlation coefficient; RR, respiratory rate, VT, tidal volume; ΔP, driving pressure

References

1. Grieco DL, Maggiore SM, Bellani G, et al. Individualized positive end-expiratory pressure guided by end-expiratory lung volume in early acute respiratory distress syndrome: study protocol for the multicenter, randomized IPERPEEP trial. *Trials.* 2022;23:63.
2. Frick AE, Schiefer J, Maleczek M, et al. The Effect of Prone Positioning After Lung Transplantation. *Ann Thorac Surg.* 2023.
3. Schwarz S, Benazzo A, Dunkler D, et al. Ventilation parameters and early graft function in double lung transplantation. *J Heart Lung Transplant.* 2021;40:4-11.
4. Veraar C, Kirschner E, Schwarz S, et al. Follistatin-like 1 and Biomarkers of Neutrophil Activation Are Associated with Poor Short-Term Outcome after Lung Transplantation on VA-ECMO. *Biology (Basel).* 2022;11.
5. Veraar C, Schwarz S, Thanner J, et al. Transient perioperative inflammation following lung transplantation and major thoracic surgery with elective extracorporeal support: a prospective observational study. *Ann Transl Med.* 2021;9:385.
6. Benazzo A, Schwarz S, Frommlet F, et al. Twenty-year experience with extracorporeal life support as bridge to lung transplantation. *J Thorac Cardiovasc Surg.* 2019;157:2515-2525 e2510.
7. Hoetzenecker K, Benazzo A, Stork T, et al. Bilateral lung transplantation on intraoperative extracorporeal membrane oxygenator: An observational study. *J Thorac Cardiovasc Surg.* 2020;160:320-327 e321.
